# Total, sex and age-specific excess mortality during 2020 in 20 countries part of the C-MOR consortium

**DOI:** 10.1093/eurpub/ckac131.044

**Published:** 2022-10-25

**Authors:** CA Demetriou, S Achilleos, A Quattrocchi, N Nicolaou, J Gabel

**Affiliations:** 1 Department of Primary Care and Population Health, University of Nicosia Medical School, Nicosia, Cyprus; 2 Department of Basic and Clinical Sciences, University of Nicosia Medical School, Nicosia, Cyprus; 3 University of Nicosia Medical School, Nicosia, Cyprus; 4 C-MOR consortium

## Abstract

Nationally published COVID-19 mortality estimates might underestimate the actual mortality burden attributed to COVID-19. Estimations of excess all-cause mortality can provide more accurate estimates of the toll of the pandemic. This study aims to estimate the overall, sex and age-specific excess all-cause mortality in 20 countries, during 2020. Total, sex and age-specific weekly all-cause death counts for 2015-2020 were extracted from national vital statistics databases. Percent excess mortality for 2020 was calculated by comparing average weekly 2020 mortality rates against average weekly mortality rates from the past five years (2015-2019). Comparisons were performed for the total population, per sex, and per age groups (<65 vs. 65+ or < 70 vs.70+) depending on data availability. Percent difference in average weekly mortality between 2020 and 2015-2019 ranged from negative for Australia and Norway, to < 5% for Denmark, Cyprus, Estonia, Israel, and Sweden, 5-10% for Georgia, Mauritius, Ukraine, Austria, France, Scotland and Northern Ireland, to ∼10-21% for England & Wales, Italy, Brazil, USA, Slovenia, and to 89% for Peru. The percent difference in average weekly mortality between 2020 and 2015-2019 for males was higher than for females except for Cyprus, Estonia, Slovenia and the USA. Lastly, in age specific analyses, for the majority of countries the % increase in average weekly mortality between 2020 and 2015-2019, was higher in the oldest age group investigated, however, for Peru and the USA (<65 vs. 65+ years) and for Cyprus and Mauritius (<70 vs. 70+ years), mortality increased similarly in both age groups. This study highlights that the excess mortality burden during the COVID-19 pandemic disproportionally affected specific countries, males, and in most, but not all countries, the oldest age groups. Strengthening of health resilience in the most affected countries, while targeting population groups impacted the most, is of paramount public health importance.

**Key messages:**

• Excess mortality burden during the COVID-19 pandemic disproportionally affected specific countries, and even within countries specific sex and age groups.

• Further investigation into the determinants of excess mortality is needed to suggest steps to strengthen health resilience in the countries and target population groups impacted the most.

